# Supplementary material for: Colloidal lead iodide nanorings
Source: Nanoscale. 2018 Nov 12;10(45):21197–208. doi: 10.1039/c8nr06430j (PMC6251342; doi:10.1039/c8nr06430j)
Supplement: Supplementary file 1 [file NR-010-C8NR06430J-s001.pdf]

Electronic Supplementary Material (ESI) for Nanoscale.
This journal is © The Royal Society of Chemistry 2018

Supporting information

Colloidal Lead Iodide Nanorings

Eugen Klein[1], Leonard Heymann[1],
Ana B. Hungria[2], Rostyslav Lesyuk[1,3], Christian Klinke[1,4,]*

[1] *Institute of Physical Chemistry, University of Hamburg, Martin-Luther-King-Platz 6, 20146 Hamburg, Germany*
[2] *Universidad de Cádiz. Facultad de Ciencias, Campus Rio San Pedro, Cadiz 11510, Spain*
[3] *Pidstryhach Institute for applied problems of mechanics and mathematics of NAS of Ukraine, Naukowa str. 3b, 79060 Lviv, Ukraine*
[4] *Department of Chemistry, Swansea University – Singleton Park, Swansea SA2 8PP, United Kingdom*

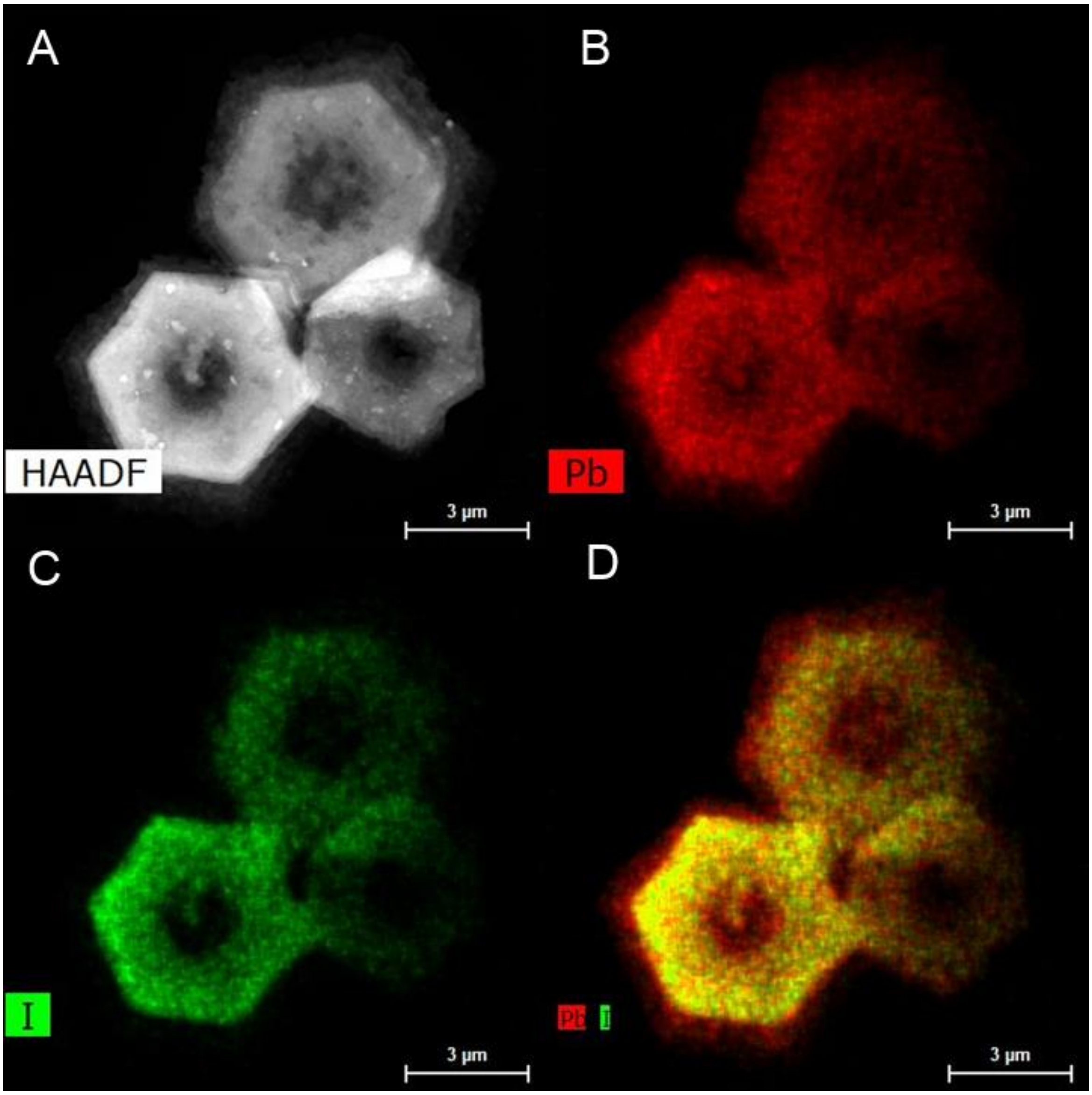

S1. (A) HAADF-STEM image of PbI_2 nanorings, (B) XEDS elemental mapping showing the spatial distribution of Pb (red), (C) I (green), and (D) Pb+I of the set of nanorings shown in panel (A). The EDS maps are deconvoluted and smoothed to improve the visualization.

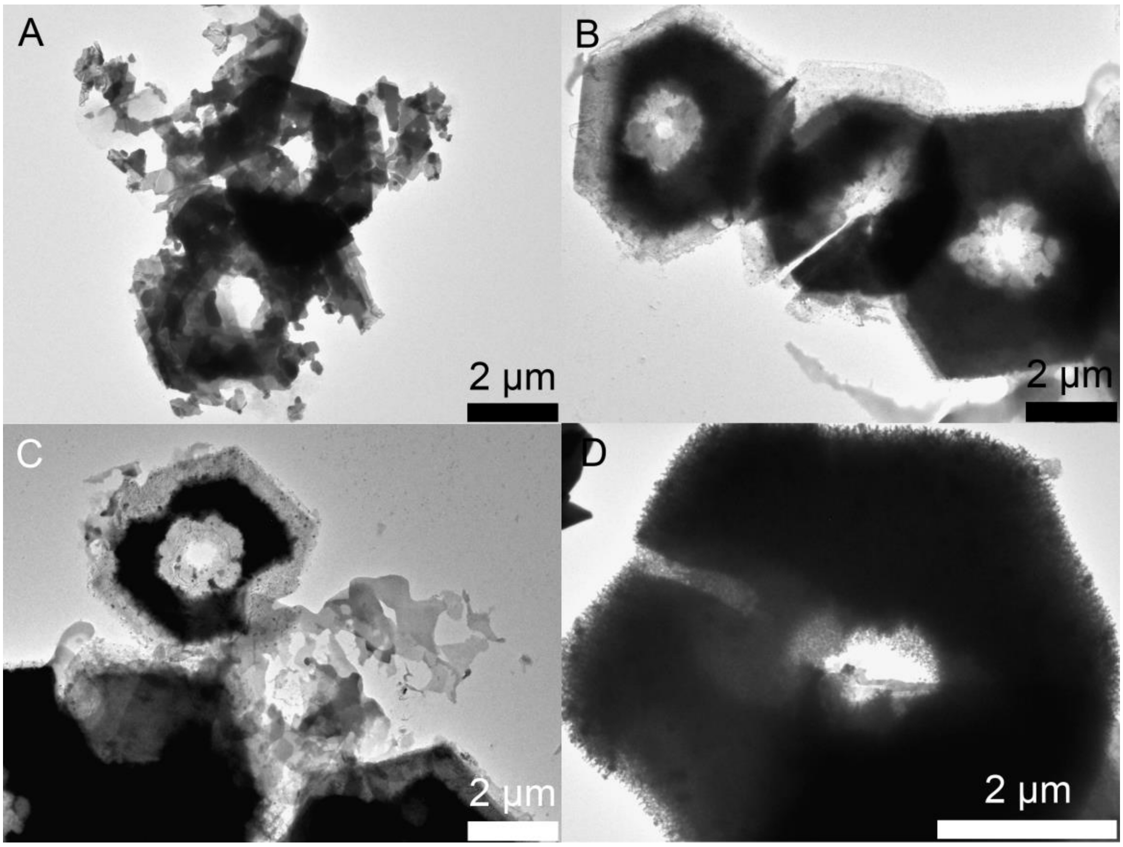

S2. TEM images of ring syntheses above 200 °C and below 100 °C. (A) PbI_2 nanosheets etched at 250 °C. (B) and (C) Preperation of ring structures at 30 °C. (D) Etching process done in a falcon tube.

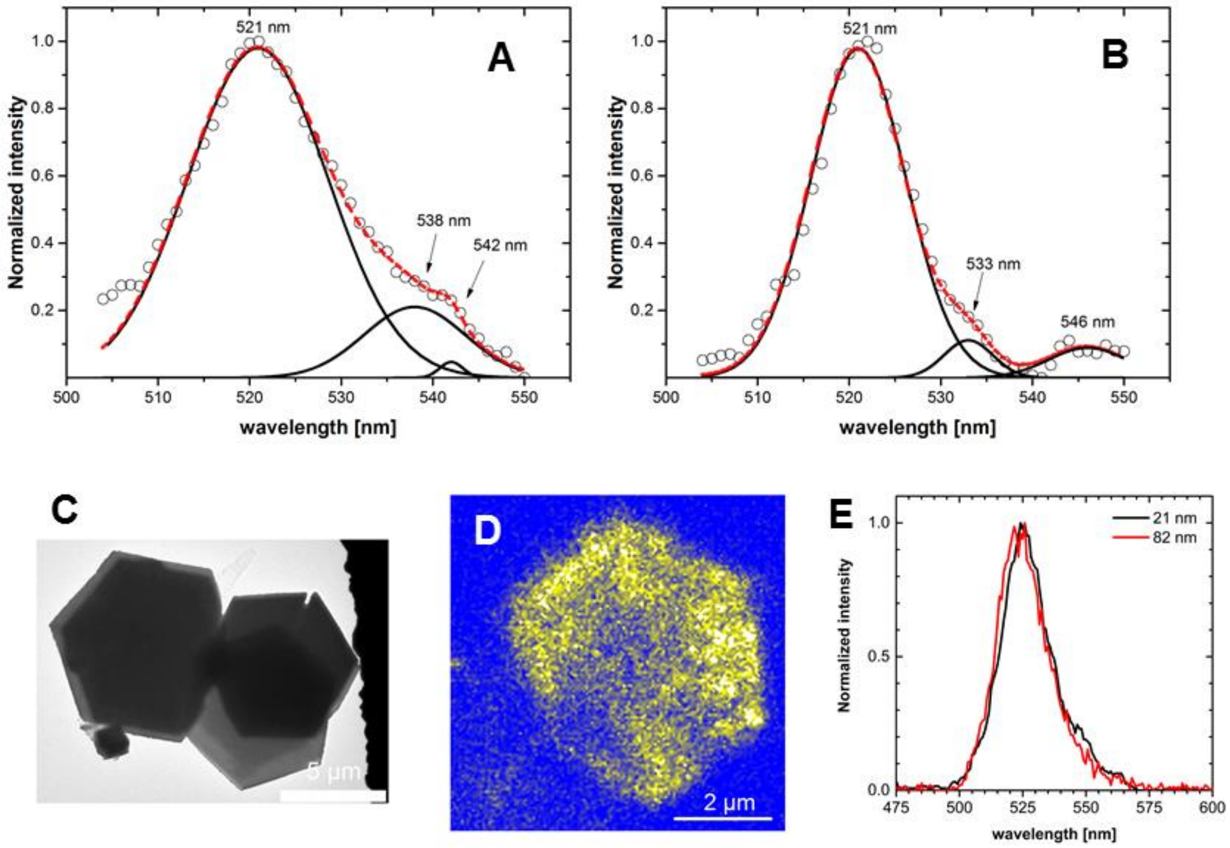

S3. (A, B) PL emission spectra of the nanorings ensembles in solution numerically fitted with Gaussian curves: (A) thin nanorings with average thickness of 21 nm; (B) thicker nanorings with average thickness of 82 nm. (C) and (D) TEM image and PL emission of unetched PbI_2 nanosheets detected by confocal microscopy. (E) PL emission of two individually picked rings on the silicon substrate from samples with different average thicknesses.

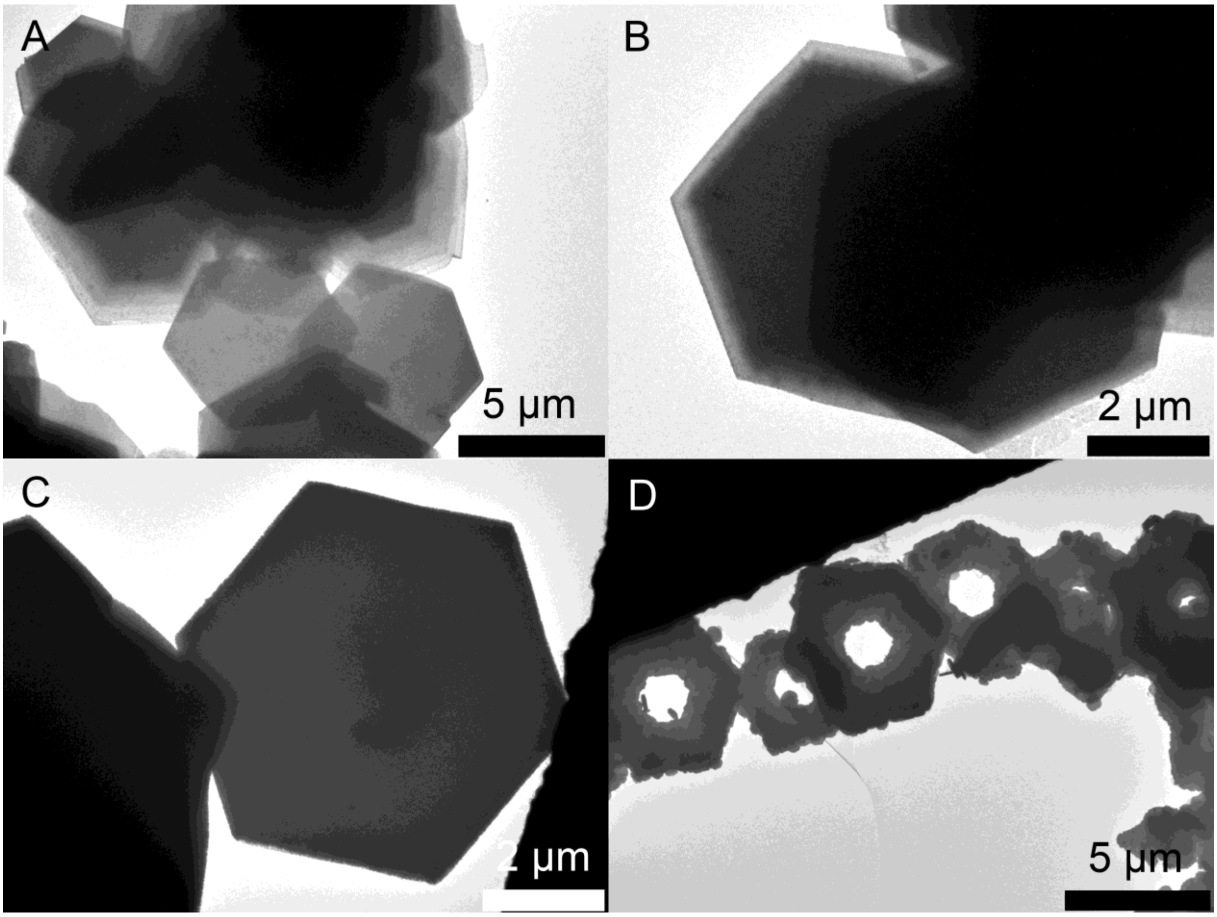

S4. TEM images of the same sample in different environments. (A) Sheets right after the synthesis. (B) The same sample after storage in a freezer for 3 days. (C) Part of the same sample diluted with oleic acid and stored for 3 days in a freezer. (D) Another part of the same sample suspended in TOP and stored for 3 days in a freezer.

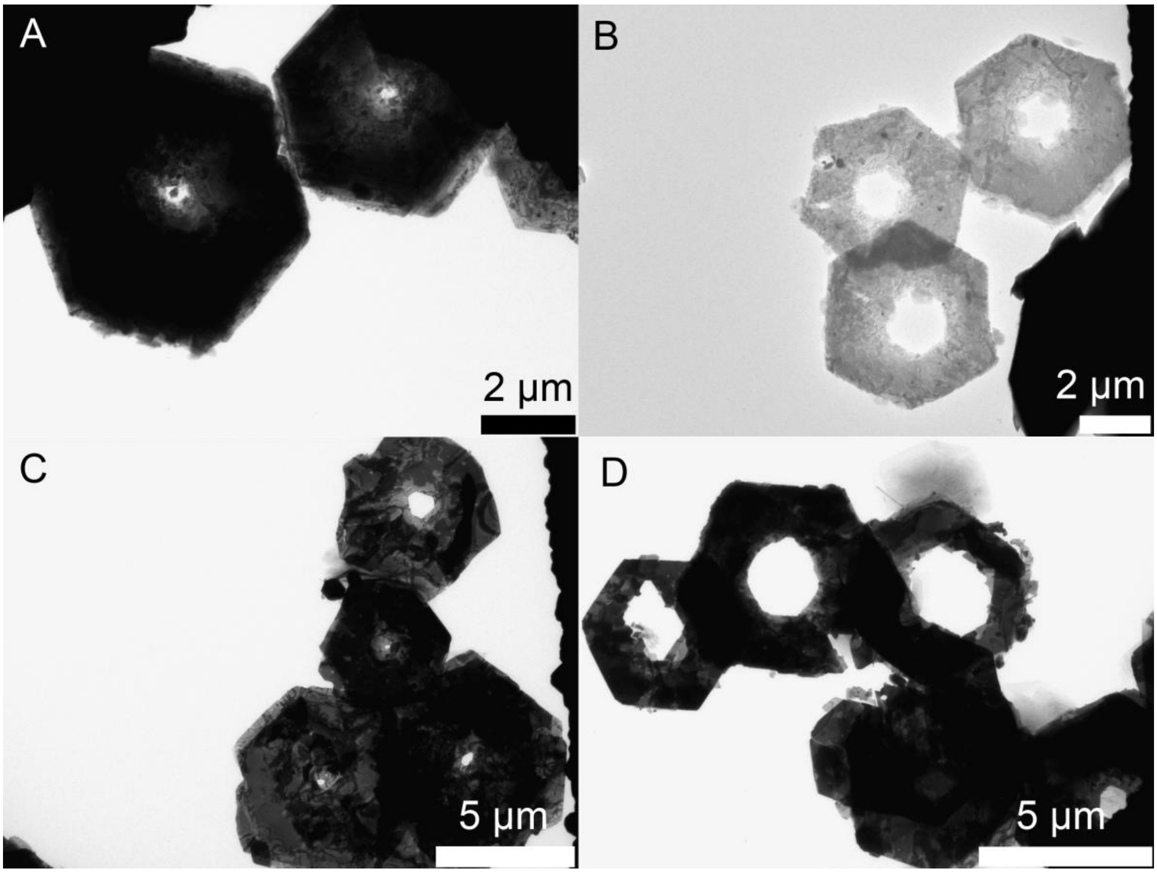

S5. (A) and (B) ring structures prepared at 100 °C with 0.08 mL and 0.12 mL of TOP. (C) and (D) ring structures prepared at 200 °C with 0.06 mL and 0.08 mL of TOP.

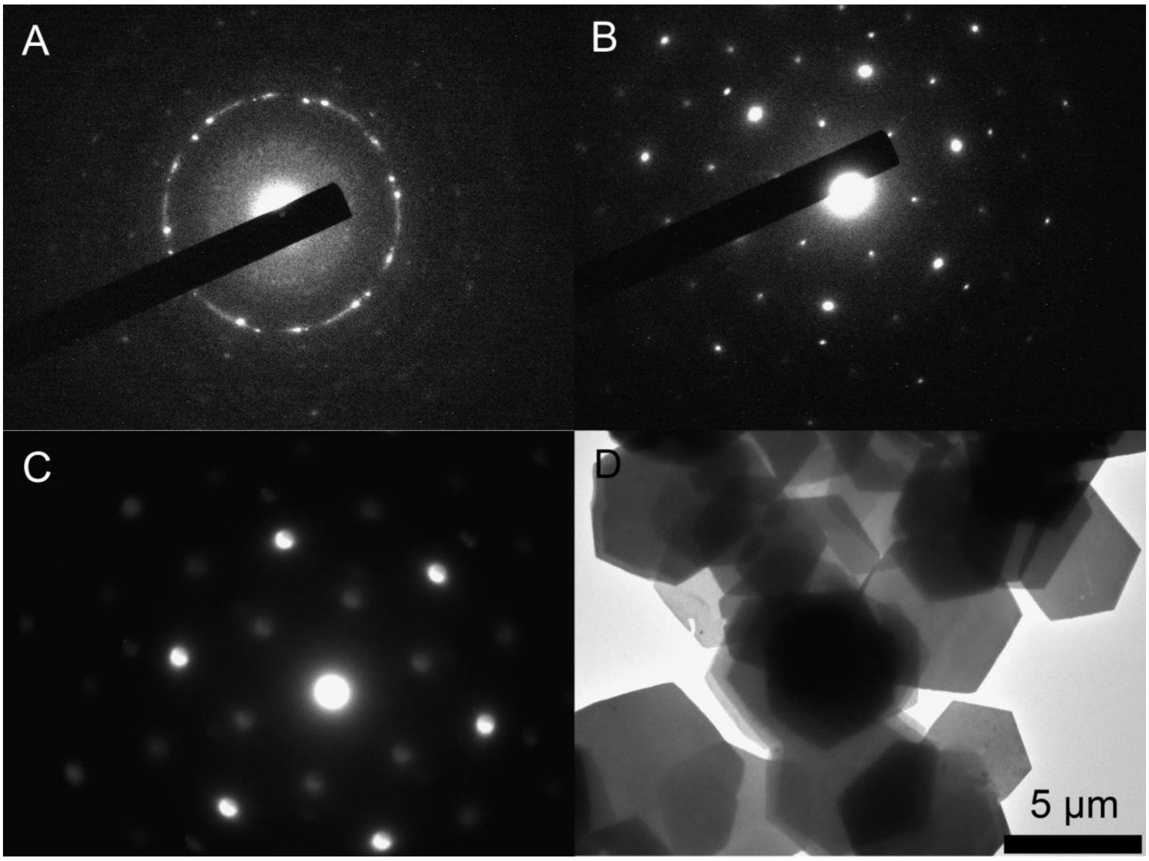

S6. (A) SAED of crystallizing PbI_2 nanosheets for the synthesis in oleic acid. (B) and (C) SAEDs of fully crystallized nanosheets prepared in oleic acid and nonanoic acid, respectively. (D) Overview for fully crystallized nanosheets prepared in nonanoic acid.

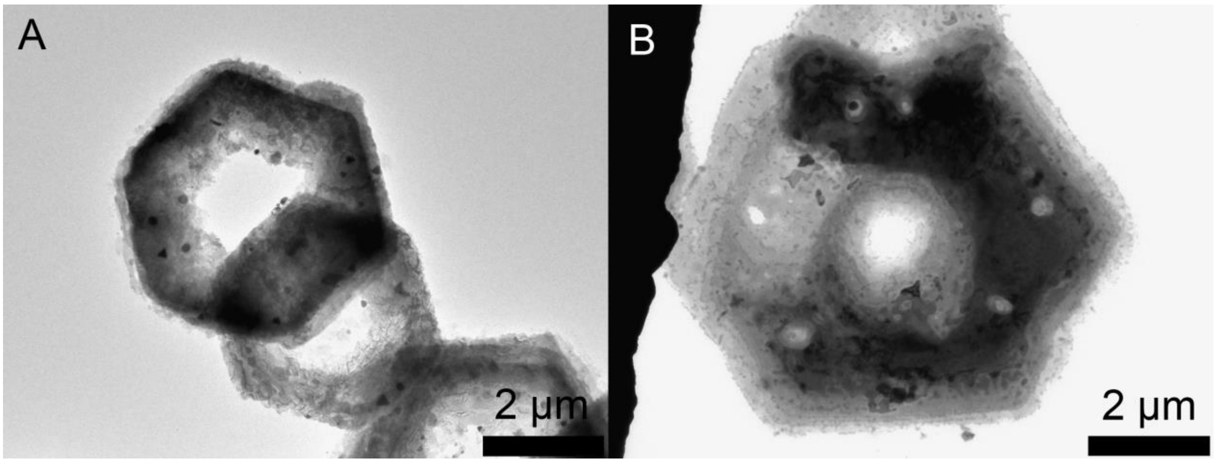

S7. Nanorings synthesized in octanoic acid (A) and in heptanoic acid (B).

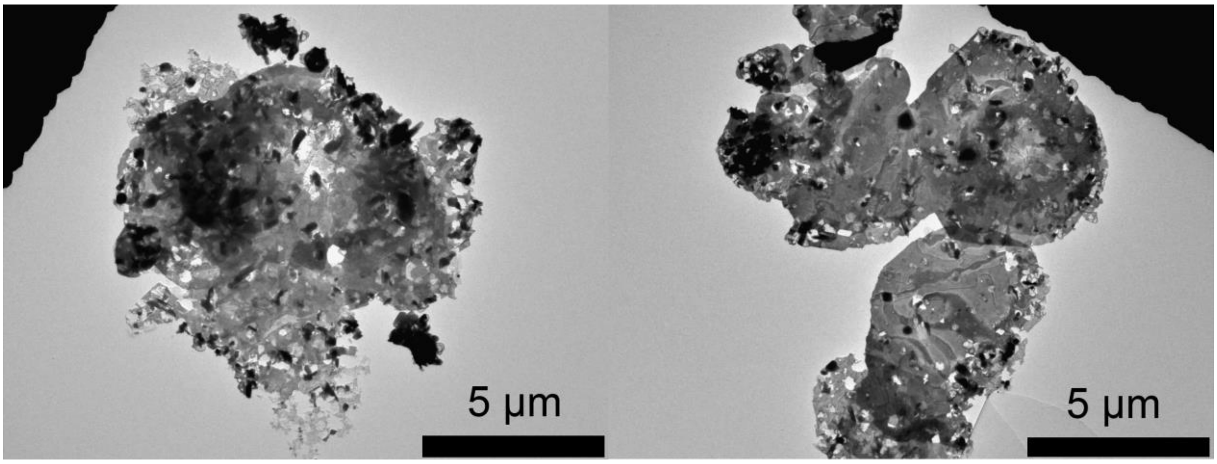

S8. TEM images of etched nanosheets which were prepared in oleic acid.

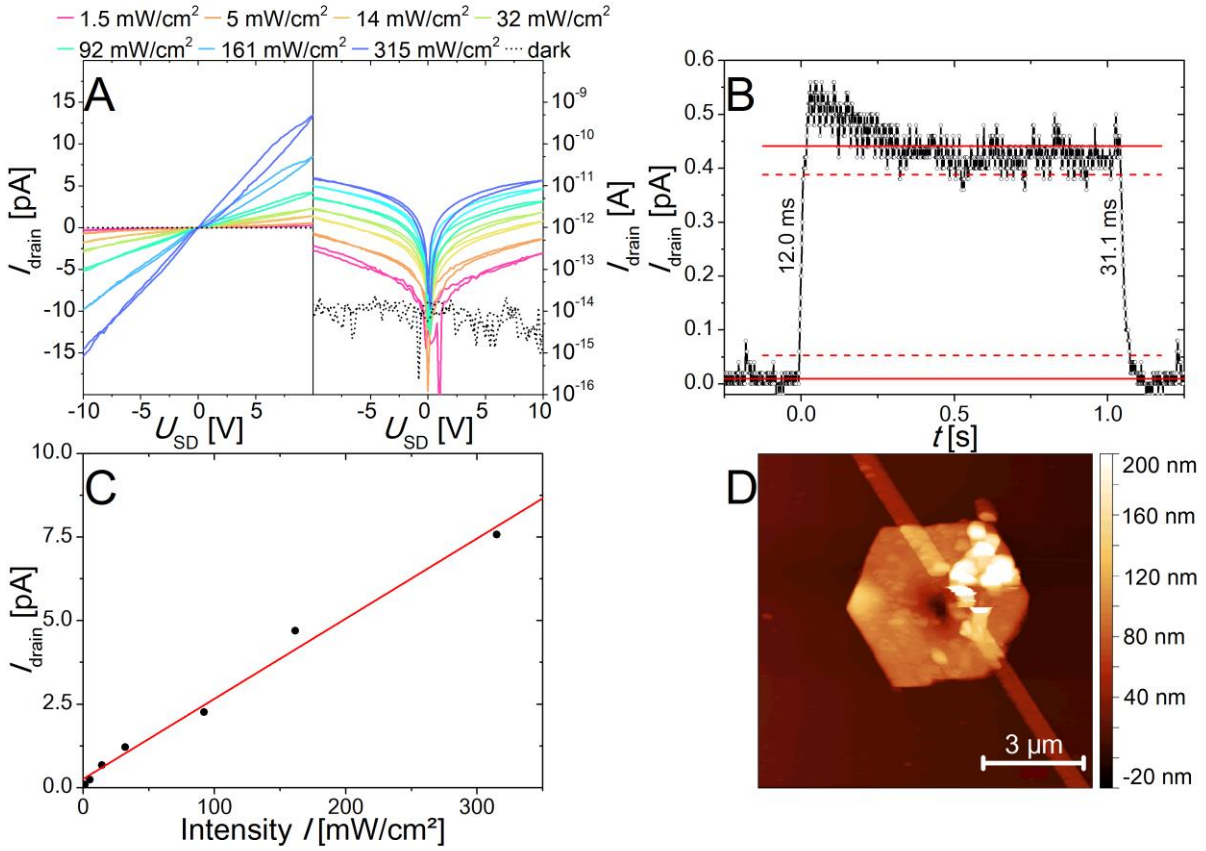

S9. IV curves of a single PbI_2 nanoring device under illumination at different intensities at 405 nm in linear scale (left) and logarithmic scale (right) (A). Evaluation of rise and fall time of PbI_2 nanorings exposed by a pulsed 405 nm laser diode with 315 mW/cm² at a frequency of 10 Hz. The red line indicates the averages of dark and photocurrent, the dashed lines 10 % and 90 % of the averaged photocurrent (B). The photocurrent at 5 V shows a linear dependency of the light power intensity used for excitation (C). AFM image of the characterized device (D).
